# Analysis of self-healing of depression by helping others in adolescents from the perspective of constructivism

**DOI:** 10.3389/fpsyt.2023.1201923

**Published:** 2023-08-03

**Authors:** Zhonggui Xin, Shuguang Li, Yanxian Jia, Hui Yuan

**Affiliations:** ^1^School of Teachers’ Education, Chaohu University, Chaohu, China; ^2^The Library, Chaohu University, Chaohu, China

**Keywords:** constructivism, adolescents, depression, helping others, self-healing

## Abstract

**Purpose:**

This study aims to explore the mechanism of psychological qualities constructed in helping others with depression and guide adolescents to actively participate in the practical activities of helping others to prevent and self-heal depression.

**Method:**

Symptom self-rating scale, trait coping style questionnaire, and self-administered helper scale were employed. A total of 1,086 valid on-site questionnaires were collected from adolescents.

**Result:**

The depression levels of adolescents were negatively correlated with helping beliefs, behaviors and total scores (*r* = −0.500, −0.401, and −0.530). Helping others had a significantly negative predictive effect on depression, effectively inhibiting depression levels. Although the positive coping style had an inhibitory effect on depression, it exerted no predictive effects on depression under the influence of helping others. In contrast, the negative coping style had a significantly positive predictive effect on depression.

**Conclusion:**

Proactively participating in helping others is an important way to prevent and eliminate depression in adolescents. They should be instructed to give full play to their initiatives to participate in social practice and assist others actively, thus constructing positive psychological qualities, improving mental health, and achieving self-healing of depression and self-help through helping others.

## 1. Introduction

Constructivism emphasizes that knowledge is not passively received but actively constructed (1), and it can only be actively constructed by individuals and internalized into their knowledge system or life experience instead of being transferred from the mind of teachers to that of students (2). Similarly, the mental health of a person is constructed by their practice, comprising creative and harmonious thinking abilities when solving practical problems in life, and is the healthy self-created through personal participation, interactions, and behavioral feedback (3, 4). However, the positive impact of practical activities on the mental health of adolescents is not valued, especially for the treatment of serious mental health problems such as anxiety and depression. People often only focus on the effect of medication (5–7) and neglect the role of psychological counseling and self-construction. Psychiatric patients are increasingly vulnerable when hospitalized and living in the community and engaging in interpersonal interactions are helpful for emotional regulation (8). Psychotropic drugs are reportedly damaging to the hearts of patients (9, 10), and medical medication increases the risk of sudden cardiac death in psychiatric patients (11). Medication alone cannot eradicate depression (12, 13) and performs poorly (14). There is no panacea for depression, and the fundamental way to eliminate depression requires the self-reconstruction of a positive psychological system (15). Helping others is important for individuals to construct a positive psychological system through interactions with people. Psychological counseling emphasizes “helping people and helping themselves” (16), which means that the dedicator achieves self-help by helping the recipient to learn to solve problems (17–19). In other words, in offering help to others, the inner emotional experience of the helpers is enriched, their aspirations are satisfied, and joy and happiness are gained; additionally, the helpers sublimate their spiritual realm, enrich their minds, and improve the way to deal with setbacks and difficulties and the ability to solve problems (20, 21). This idea is consistent with that advocated by constructivism to realize the development of individual knowledge systems, mental health and ability through self-construction (22, 23). Kant also proposed that human knowledge and psychological qualities were constructed by combining intellectual categories and perceptual experience. Without subject construction, the formation of knowledge and psychological qualities was not possible [(24), p. 7]. Such proposals as discovery learning by Bruner [(25), p. 5], generative cognition theory by Piaget [(26), p. 17], and cooperative learning and educational practice by Vygorsky [(27), pp. 82–83] reflect that human knowledge and psychological qualities are constructed through practice. Only by actively participating in activities with social significance can individual behavioral patterns and knowledge systems be constructed [(28), p. 355], and helping others fully meets this requirement. In helping others, people construct their positive mentality and reduce depressive mood through experience [y3–(29), y4–(30)], and thus promote the self-healing of depression. Studies noted that mutual help effectively addressed mental health and addiction problems (31). Considering that helping others is an altruistic behavior, people with high altruistic behaviors are mentally healthier than those with low altruistic behaviors (32). Put differently, helping others willingly is a manifestation of mental health and is recognized as an important way to cultivate a healthy personality. People with a high propensity to help others show high life satisfaction and few problem behaviors. People who are willing to provide help can quickly shorten their distance from others, establish harmonious interpersonal relationships (33), be easily accepted by others, and perceive interpersonal relationships with an increasingly positive mindset (34); the mindset, in turn, has an impact on the different coping styles individuals choose (35, 36). Moreover, another factor also draws attention to adolescents’ psychological development. Studies showed that cell phone use can influence their social and emotional development (37, 38). Excessive use of social media platforms and video games can lead to feelings of anxiety, depression, and loneliness (39–41). Cyberbullying can have severe psychological consequences. The use of cell phones before bedtime can lead to sleep deprivation, which results in a negative impact on adolescents’ mental health, cognitive functioning, and overall well-being (42–44). Cell phone addiction may make them experience withdrawal symptoms, anxiety, and irritability when they are separated from their phones (45, 46). In a nutshell, cell phone use among adolescents can reduce their face-to-face communication, which makes them struggle with interpersonal interactions, empathy and effective communication techniques. In addition, several studies found that crucial factors such as gender, study pressure, and family background can affect adolescents’ depression levels. This study hypothesized that the levels of depression are negatively correlated with the helpfulness and positive coping styles; helping others and positive coping styles can effectively reduce or negatively predict depression levels.

## 2. Research method

### 2.1. Research tools

The coping style questionnaire, developed by Qianjin Jiang, contains 20 items of positive coping (PC) and negative coping (NC) dimensions. The scale ranges from 1 to 5 (1 not; 2 generally not; 3 not sure; 4 generally yes; 5 definitely yes). PC > 40 indicates that the individual adopts a positive coping style, and NC > 35 implies a negative coping style. The retest reliability of the two dimensions was 0.75 and 0.65, respectively; the correlation coefficients of PC and NC between the subjects and the family test were 0.75 and 0.73, respectively (47). The study showed a Cronbach’s alpha coefficient of 0.78 for the coping style questionnaire total scale.

The symptom self-rating scale, designed by Derogatis and Savitz (48), includes 90 items, and only depressive symptoms were analyzed in this study. The questionnaire was a five-point scale (1 = never, 2 = mild, 3 = moderate, 4 = quite severe, and 5 = severe). Any factor score reaching 2 indicated a positive item (48).

The help scale was devised using a self-administered 5-point Likert scale (1 = very unsuitable, 2 = not very suitable, 3 = uncertain, 4 = quite suitable, 5 = very suitable). In accordance with the requirements and procedures of the scale, 12 items were designed to predict 425 adolescents, and 9 items were retained after analysis. Two dimensions were determined and named “helping beliefs” and “helping behaviors.” The former comprised perceptions and attitudes toward helping others, and the latter constituted behavioral activities that benefit or help others. After the scale was performed, 1,086 adolescents were surveyed. It was revealed that the eigenvalues of the two dimensions were 4.193 and 1.524, respectively, and the contribution rates were 46.592 and 16.934%, respectively, with a cumulative contribution rate of 63.526%. The higher scale score represents a higher propensity to help people; those with a median score of 27 or less have a low propensity to help others, and those with a score of 36 or more show a high propensity to help people. The Kaiser-Meyer-Olkim test and Bartlett’s sphericity test was conducted on the 9-item scale to examine the appropriateness of the factor analysis of “helping people.” The results showed that the Kaiser-Meyer-Olkim coefficient was 0.858 > 0.5, indicating relatively large common factors among the variables; the chi-square value of Bartlett’s sphericity test was 4169.115, with a degree of freedom of 36 and a significance of 0.000 < 0.05 (at a significance level), indicating that common factors exist among the items and the sample is suitable for factor analysis. The reliability and validity were verified to be relatively good. The final internal consistency coefficient (Cronbach’s alpha coefficient) was 0.852, and the coefficients of the two dimensions were 0.860 and 0.786, respectively. The specific factor analysis is shown in Table 1.

**Table 1:** Detailed analysis of factors on the help scale.

Dimension name	Question number and item	Factor 1	Factor 2	Common degree
Helping beliefs	T1, I believe in giving roses and leaving fragrance in my hands	0.838		0.734
	T2, I think people need to help each other	0.834		0.712
	T9, I believe helping others is one of the most attractive qualities in a person T4, I believe that to help others is to broaden the road for yourself	0.832 0.716		0.747 0.561
	T3, I always trust others	0.669		0.480
Helping behaviors	T6, I often participate in volunteer services or peer support activities		0.873	0.775
	T7, It’s always a pleasure to help others		0.772	0.619
	T5, I often help my classmates to do what I can		0.749	0.602
	T8, I always donate some pocket money to events that require donations		0.629	0.487
	Eigenvalue	4.193	1.524	
	Contribution rate (%)	46.592	16.934	
	Cumulative contribution rate (%)	46.592	63.526	

### 2.2. Research object

According to the survey design, from September 1 to 30, 2021, subjects were selected in several middle schools and universities in Hefei, Anhui Province, and the survey was implemented in the classroom with the assistance of mental health education teachers. A total of 1,086 valid questionnaires were collected, including 531 middle school students (270 junior middle school students and 261 high school students) and 555 college students (258 sophomores and 297 juniors). There were 480 male students (44.2%), 606 and female students (55.8%). Among them, 387 students (35.6%) came from urban areas and 699 (64.4%) from rural areas. Regarding the mobile phone use duration, four types were divided: within 1 h, 1–2 h, 2–3 h, and more than 3 h. The number of people using mobile phones within 1 h and more than 3 h is relatively small, mainly concentrated between 1 and 3 h in a normal distribution, and thus the median value was taken. The mobile phone use time was categorized into two parts: within 2 h and more than 2 h. A total of 411 students used mobile phones within 2 h, accounting for 37.4%, and 675 utilized their phones for more than 2 h, contributing to 62.2% (Table 2).

**Table 2:** Sample composition distribution.

Classification	Standard	Number (*N*)	Percentage (%)
Gender	Male	480	44.2
	Female	606	55.8
Grade	Middle school	531	48.9
	University	555	51.1
Student source	Rural area	699	64.4
	Urban area	387	35.6
Mobile phone usage time	Within 2 h	411	37.8
	More than 2 h	675	62.2

## 3. Results

### 3.1. Differences in depression levels, helping others, and coping styles

Analysis of variance showed significant differences between genders in helping beliefs and positive coping styles. Female students had significantly higher helping beliefs and more positive coping styles than male students, and no differences were found in other aspects. Concerning grades, helping beliefs did not differ, while depression levels, helping behaviors, and coping styles differed significantly. Specifically, college students were more inclined to choose positive coping styles than middle school students, and they exhibited higher helping behaviors and tendencies and significantly lower depression levels than those of middle school students. The details are depicted in Table 3.

**Table 3:** Differences in depression levels, helping others and coping styles among adolescents (*M* ± SD).

	Gender		*t*	*p*	Grade		*t*	*p*
	Male *N* = 480	Female *N* = 606			Middle school *N* = 531	University *N* = 555		
A	1.528 ± 0.595	1.546 ± 0.518	−0.524	0.600	1.587 ± 0.601	1.491 ± 0.499	2.821	0.005
B	19.319 ± 3.709	19.762 ± 3.513	−2.017	0.044	19.446 ± 3.812	19.681 ± 3.393	−1.070	0.284
C	14.394 ± 3.138	14.446 ± 3.114	−0.271	0.786	13.853 ± 3.407	14.968 ± 2.720	−5.942	0.000
D	33.713 ± 5.883	34.208 ± 5.655	−1.408	0.159	33.299 ± 6.249	34.649 ± 5.169	−3.868	0.000
E	34.069 ± 4.980	34.936 ± 5.462	−2.700	0.007	34.175 ± 5.545	34.914 ± 4.971	−2.307	0.021
F	28.431 ± 6.380	28.767 ± 5.795	−0.908	0.370	29.655 ± 5.987	27.627 ± 5.968	5.590	0.000

	Mobile phone use duration		*t*	*p*	Student source		*t*	*p*
	Within 2 h *N* = 411	More than 2 h *N* = 675			Urban area *N* = 387	Rural area *N* = 699		
A	1.427 ± 0.587	1.606 ± 0.520	−5.220	0.000	1.614 ± 0.608	1.497 ± 0.516	3.204	0.001
B	19.971 ± 3.556	19.320 ± 3.614	2.896	0.004	19.504 ± 3.757	19.601 ± 3.519	−0.424	0.671
C	15.299 ± 3.097	13.889 ± 3.019	7.393	0.000	13.597 ± 3.395	14.880 ± 2.864	−6.296	0.000
D	35.270 ± 5.842	33.209 ± 5.570	5.805	0.000	33.101 ± 6.261	34.481 ± 5.405	−3.648	0.000
E	35.015 ± 5.138	34.271 ± 5.332	2.259	0.024	34.279 ± 5.357	34.704 ± 5.219	−1.273	0.203
F	26.854 ± 6.202	29.693 ± 5.715	−7.535	0.000	29.364 ± 6.309	28.206 ± 5.881	3.028	0.003

A, depression; B, helping beliefs; C, helping behaviors; D, helping total score; E, positive coping; F, negative coping; same below.

Concerning the duration of mobile phone use, participants who used mobile phones for more than 2 h showed high levels of depression and were inclined to choose negative coping styles. In contrast, those utilizing mobile phones for less than 2 h displayed lower levels of depression, more helping beliefs and behaviors, and higher tendencies to help others and choose positive coping styles. Excerpt for helping beliefs and positive coping styles among students of different sources, significant differences were observed in depression levels, helping behaviors, tendencies to help people and negative coping styles, indicating that urban students have higher levels of depression and more tendencies to negative coping styles than rural students, and significantly lower helping behaviors and tendencies than rural students.

### 3.2. Correlation of depression levels, helping others, and coping styles

As shown in Table 4, depression levels were significantly and negatively correlated with helping beliefs, behaviors and tendencies and positive coping styles (*r* = 0.500, −0.401, −0.530, and −0.347, respectively), and significantly and positively correlated with negative coping styles (*r* = 0.494). The positive coping style was significantly and positively correlated with helping beliefs and behaviors, and the total helping score (*r* = 0.386–0.582), and the negative coping style was significantly and negatively correlated with the three helping indicators (*r* = −0.349 to −0.421). An increase in helping tendencies was consistent with a rise in positive coping styles and the opposite direction of an increase in depression levels. In contrast, the increasing negative coping styles conformed with the high depression levels and oriented oppositely with the upgrading helping tendencies. It is reflected that helping tendencies and positive coping styles can reduce depression levels and promote mental health.

**Table 4:** Correlation coefficients of depression levels, helping indicators and coping styles among adolescents.

	A. Depression levels	B. Helping beliefs	C. Helping behaviors	D. Helping scores	E. Positive coping styles	F. Negative coping styles
A	1.000	1.000	1.000	1.000	1.000	
B	−0.500^**^	0.463^**^	0.832^**^	0.573^**^	−0.321^**^	
C	−0.401^**^	0.877^**^	0.386^**^			
D	−0.530^**^	0.582^**^				
E	−0.347^**^					
F	0.494^**^	−0.370^**^	−0.349^**^	−0.421^**^		1.000

** Indicates significant correlation at 0.01 level (both sides).

### 3.3. Regression of helping others and coping styles on depression levels in adolescents

The stepwise regression analysis with depression level as the dependent variable and helping beliefs and behaviors, and positive and negative coping styles as the independent variables showed an R2 of 0.376 and an adjusted R2 of 0.374, reflecting a high explanation degree of the predictive effect of independent variables on the dependent variable, and the regression equation is as follows:


$$Y=a+b1^{*}\mathrm{X1}+b2^{*}\mathrm{X2}+b3^{*}\mathrm{X3}+b4^{*}\mathrm{X4}+e$$

As shown in Table 5, helping beliefs and behaviors had a significant negative predictive effect on depression levels, positive coping styles had a non-significant predictive effect on depression levels, and negative coping styles had a positive predictive effect on depression levels. Regression models are developed as follows:


$$\begin{array}{l} \mathrm{Depression level}=1.975-0.048^{*}\mathrm{Helping beliefs}+0.03^{*}\\ \;\;\;\;\;\;\;\;\;\;\;\;\;\;\;\;\;\;\;\;\;\;\;\;\;\;\;\;\;\;\;\;\;\;\;\;\;\;\;\;\;\;\;\;\;\mathrm{Negative coping styles}\\ \;\;\;\;\;\;\;\;\;\;\;\;\;\;\;\;\;\;\;\;\;\;\;\;\;\;\;\;\;\;\;\;\;\;\;\;\;\;\;\;\;\;\;\;\;-0.025^{*}\mathrm{Helping behaviors} \end{array}$$

**Table 5:** Regression analysis with depression level as the dependent variable.

Dependent variable	Independent variables	*B*	*β*	*t*	*p*	VIF	*F*	p	*R* ^2^
Depression levels	Constant	1.975		14.917	0.000		216.862	0.000	0.376
	Helping beliefs	−0.048	−0.312	−11.160	0.000	1.358			
	Negative coping styles	0.030	0.330	12.451	0.000	1.215			
	Helping behaviors	−0.025	−0.141	−5.069	0.000	1.335			

It can be seen from the regression model that helping behaviors and beliefs had a negative predictive effect on depression levels, significantly inhibiting the increase in depression. Specifically, each increase of one variable in helping beliefs decreased depression levels by a 0.048 variable, and each additional variable in helping behaviors reduced depression levels by a 0.025 variable. Furthermore, helping beliefs had a more significant inhibitory effect on depression than helping behaviors. In contrast, the negative coping style had a positive predictive effect on depression levels, with each increase elevating depression levels by a 0.03 variable.

### 3.4. The regulation of the effect of coping styles on depression by helping tendencies

**Figure 1:**
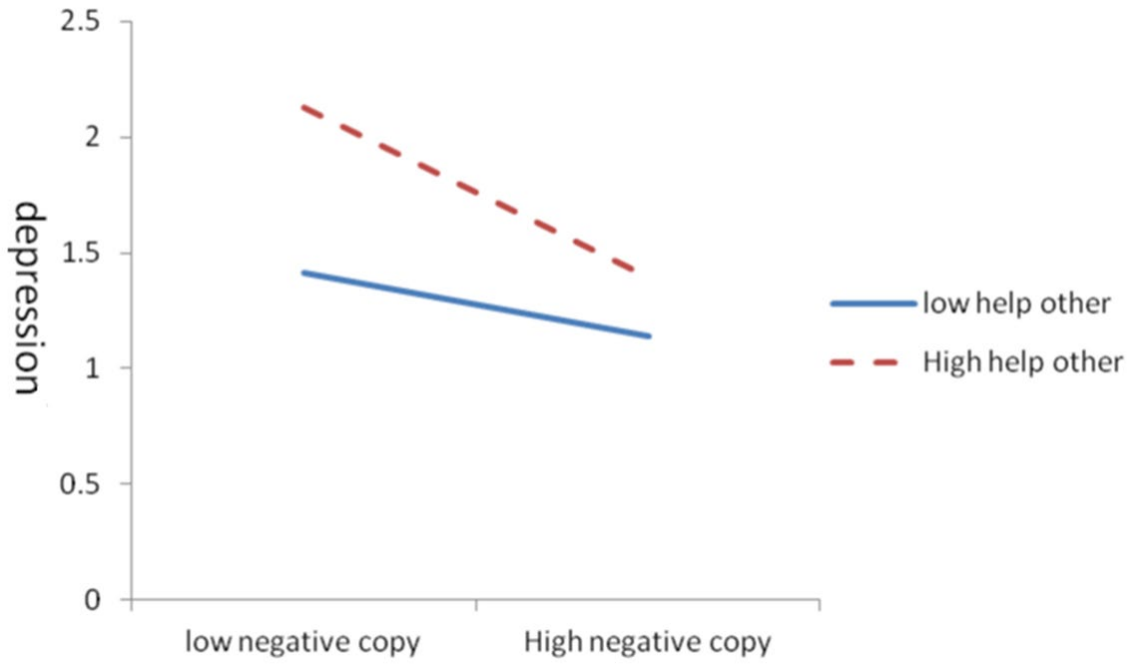
Moderation of the effect of negative coping styles on depression by helping others.

**Figure 2:**
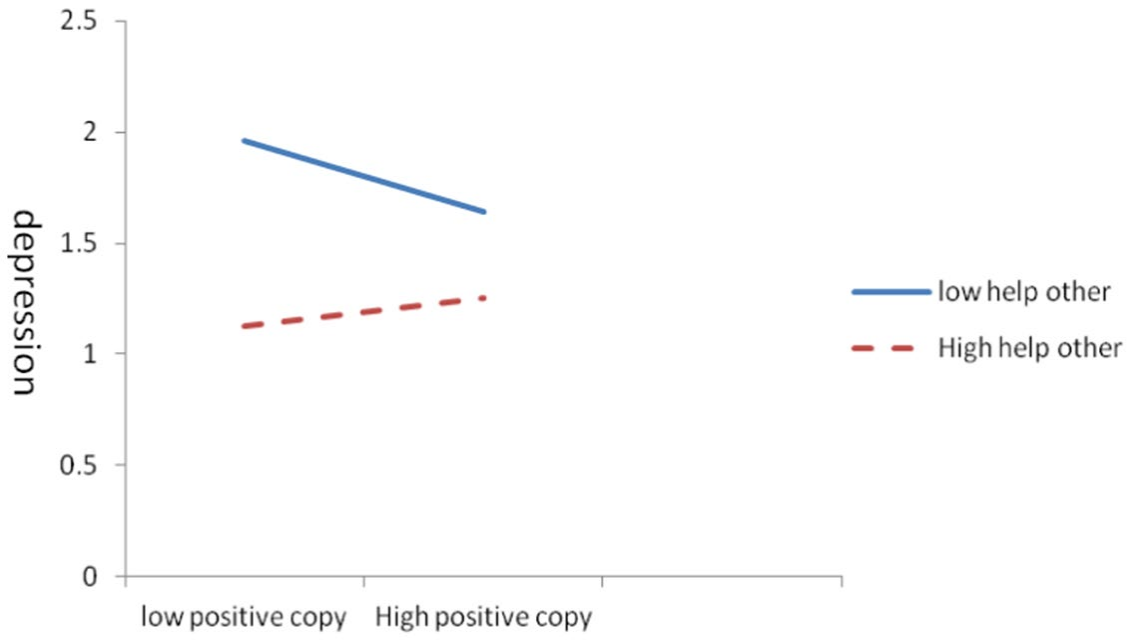
Moderation of the effect of positive coping styles on depression by helping others.

In the influence of coping styles on depression levels, helping tendencies exhibited a certain moderating effect. As shown in Table 6, the effect of negative coping styles on depression levels was moderated by helping tendencies, the moderating effect was −0.002, and R2 was 0.03. Figure 1 showed that under negative coping styles, the higher tendencies to help others suggested the faster depression reduction. In other words, in the case of negative coping styles, increasing the tendency to help others is conducive to improving mental health, realizing the elimination and self-healing of depressive disorder. The influence of positive coping styles on depression levels was moderated by helping tendencies, the moderating effect was 0.003, and R2 was 0.046. Figure 2 demonstrated that in the positive coping state, low helping tendencies led to a rapid decline in depression levels, and high helping tendencies slowly increased depression levels.

**Table 6:** The regulation of depressive mood by helping others.

Dependent	Independent	*β*	*t*	*R* ^2^	Δ*R*^2^	*F*
Depression levels	Negative coping styles	0.030	12.845	0.401	0.030	54.614**
	Helping others	−0.035	−14.117			
	Help*negative	−0.002	−7.390			
	Positive coping styles	−0.008	−2.467	0.330	0.046	74.879**
	Helping others	−0.042	−14.186			
	Help* positive	0.003	8.653			

** Indicates significant correlation at 0.01 level (both sides).

## 4. Analysis and discussion

According to the results, the levels of depression in adolescents were not influenced by gender. Previous studies have shown that women suffered from mental illness more than men (8), yet gender differences may not appear in depression levels during adolescence. This may be related to the growth and educational experiences of the adolescents. The learning tasks and family and social responsibilities undertaken by adolescents are no longer differentiated by gender, and the environment and requirements for their growth are highly consistent. In this sense, gender differences may simply be misunderstood (49).

In terms of educational levels, middle school students had higher levels of depression and were more prone to negative coping styles than college students, and college students were more likely to choose positive coping styles than middle school students. College students had a higher propensity to help others with significantly more helping behaviors than middle school students. Grade differences were not detected in helping beliefs between the two groups, indicating that middle school students also have high helping ideas and beliefs. Due to the limited learning environment and external conditions, they cannot fully participate in practice, resulting in restricted helping behaviors. In contrast, college students have more time and energy to devote to helping behaviors, participate in more social practices, are more capable of dealing with psychological confusion and stress, and have lower levels of depression. Research has pointed out that participating in practical activities, interpersonal communication, and collective or interactive tasks can improve the positive mentality and reduce the “Buddha-like mentality” (50). College students have more freedom than middle school students in their studies since their academic burdens and pressure are relatively small. These factors may affect the difference in helping behaviors and depression levels among adolescents in different grades.

Teenagers who spent more time using mobile phones had higher levels of depression, more negative coping styles, fewer helping behaviors, and smaller tendencies to positive coping styles. This is consistent with the studies that the longer mobile phone use time of adolescents indicated higher anxiety levels, lower verbal adequacy, and more serious verbal exhaustion (51). In other words, adolescents devoting more time to the virtual world inevitably reduce the behavior of helping others and interpersonal interactions, thus lacking the opportunities to gain pleasure in practice and having difficulties alleviating depression (52). Although cell phones bring convenience to people, they also inhibit adolescents from social participation and development and self-construction of psychological qualities, reflecting the importance of helping others and active participation in social practices for constructing positive psychological qualities and self-healing psychological problems.

The depression levels of urban adolescents were significantly higher than those of rural adolescents, the helping behaviors and tendencies were significantly lower in urban adolescents than those in rural adolescents, and urban adolescents were more inclined to choose negative coping styles. It is reflected that the freer environment of rural areas is more suitable for the healthy growth of adolescents, allowing for more face-to-face contact, practical exercises, and opportunities to be involved in helping others and interpersonal interactions, thus constructing positive ideas through their own experiences. Positive psychological qualities promote individuals to easily internalize knowledge or information into their cognitive system (53), thereby changing their own cognitive and behavioral results (54), and effectively promoting the prevention and self-healing of depression.

The depression levels, helping tendencies, and positive coping styles of adolescents were mutually inhibited, while depression and negative coping styles were mutually promoted. The tendency to help others can promote the development of positive psychological qualities and active coping manners towards adversity, thus establishing a buffer between events and physical and mental reactions when encountering negative stimuli (55). Additionally, the goal of reducing depression can be achieved as a result of improved mental health and coping manners with life by helping others. The result agrees with previous studies which pointed out that helping others could arouse the biological mechanism of the social perception of individuals and promote them to actively deal with events in life (56). Participating in practical activities with a positive attitude was argued to promote the formation and development of positive psychological qualities and change the subconscious cognition and positive psychological experience of participants (57, 58). A positive attitude also contributed to the therapeutic effect of major diseases (59). In this sense, guiding young people to proactively participate in voluntary services and helping activities can promote them to devote themselves to practical activities and interpersonal interactions, thus enhancing their abilities to learn cooperation and mutual assistance, construct positive psychological qualities, and face various challenges and difficulties with a positive attitude and coping style.

Helping people and depression had two-way predictive effects, helping beliefs and behaviors had significantly negative predictive effects on depression, and negative coping styles had a positive predictive effect on depression. Although the changes in helping others exerted a small negative predictive effect on depression, this prediction was still significant, and depression was effectively suppressed by helping others. The negative coping style can increase depression levels, and its enhancement effect on depression was much greater than the inhibitory effect of the positive coping style on depression. Individuals in a depressive state are inclined to choose an avoidant coping style (60). In contrast, adolescents interested in helping others are more likely to adopt positive coping styles, and the positive psychological qualities they develop in the process play a decisive role in their mental health. Helping others promotes the self-construction of positive psychological qualities in adolescents (61). In addition, it manifests individual integration and harmony with the collective (62) and is regarded as an important way of constructing interpersonal alliances and cooperation (63), and self-construction of healthy psychological qualities from the perspectives of interpersonal relationships and social adaptation. The construction of these positive psychological qualities has significance for the self-healing of depression.

Helping tendencies had a moderating impact on the effect of coping styles on depression; whether using positive or negative coping styles, the depression levels can be reduced to some extent with helping tendencies. In the case of a negative coping state, high helping tendencies were helpful to reduce depression, indicating that actively helping others or public welfare social activities is beneficial to improve mental health. Whereas for adolescents with a positive coping state, a low helping tendency contributed to reduced depression, and a high helping tendency slowly increased levels of depression. Helping others is a manifestation of peer support, which improves mental health (64), in people with mental illness, thereby inhibiting and eliminating depression or achieving self-healing of depression. Notably, being excessively involved in actively helping activities is not recommended to avoid overwhelming burdens.

## 5. Conclusion

In the prevention and treatment of depression, medication alone may not necessarily be enough for depression. The positive psychological qualities through helping others and practical activities are key to self-healing of depression. Helping people can be beneficial in alleviating depression. People can construct the ability for social engagement, mental resilience, and sound interpersonal skills in the process, effectively reducing the occurrence of depression. Consultation and social work emphasize “helping others is self-help.” For one thing, as the old Chinese saying goes “teaching people to fish is a lifetime benefit”; for another, the giver achieves self-help by helping others. In offering help to others, young people enrich their inner emotional experience, sublimate their spiritual realm, and construct positive psychological qualities, thus forming immunity against depression, which is consistent with the proverb “the only way to help oneself is to help others.” In the devoting process, adolescents acquire the ability to cope with difficulties and frustrations, positive self-efficacy, lasting inner happiness, the skills to serve others and deal with their interests and cooperate with others, and the virtues and self-healing of depression. According to the constructivist view on knowledge acquisition and healthy psychological formation, instructing teenagers to proactively participate in helping others, doing something useful for their classmates, and serving society to their best will help develop their abilities to help others and inspire their positive psychological qualities of integrating into the group and undertaking social responsibility, and suppress the negative effects of depression and realize self-healing of depression in the process.

## Data availability statement

The raw data supporting the conclusions of this article will be made available by the authors, without undue reservation.

## Ethics statement

The studies involving human participants were reviewed and approved by Chaohu University Teacher Education College, Academic Committee. Written informed consent to participate in this study was provided by the participants’ legal guardian/next of kin.

## Author contributions

YJ and HY: study design. ZX: collection, analyses, interpretation of data, and draft of the manuscript. SL: critical revision of the manuscript. All authors contributed to the article and approved the submitted version.

## Funding

This study was sponsored by the Anhui Provincial Key Project of Humanities and Social Sciences (SK2019A0546), the Anhui Provincial University Outstanding Talent Cultivation Program (gxyqZD2020101), the Subject Construction Promotion Project of Anhui Province (kj20fdzy05), and the Social Science Innovation Development Project of Anhui Province (2021CX195).

## Conflict of interest

The authors declare that the research was conducted in the absence of any commercial or financial relationships that could be construed as a potential conflict of interest.

## Publisher’s note

All claims expressed in this article are solely those of the authors and do not necessarily represent those of their affiliated organizations, or those of the publisher, the editors and the reviewers. Any product that may be evaluated in this article, or claim that may be made by its manufacturer, is not guaranteed or endorsed by the publisher.
